# Frequency, Spectrum, and Stability of Leaf Mutants Induced by Diverse γ-Ray Treatments in Two *Cymbidium* Hybrids

**DOI:** 10.3390/plants9040546

**Published:** 2020-04-23

**Authors:** Sang Hoon Kim, Se Won Kim, Joon-Woo Ahn, Jaihyunk Ryu, Soon-Jae Kwon, Byoung-Cheorl Kang, Jin-Baek Kim

**Affiliations:** 1 Advanced Radiation Technology Institute, Korea Atomic Energy Research Institute, Jeongeup 56212, Korea; shkim80@kaeri.re.kr (S.H.K.); sewonk@korea.kr (S.W.K.); joon@kaeri.re.kr (J.-W.A.); jhryu@kaeri.re.kr (J.R.); soonjaekwon@kaeri.re.kr (S.-J.K.); 2 National Institute of Agricultural Sciences, Rural Development Administration, Jeonju 54874, Korea; 3 Department of Plant Science, Plant Genomics and Breeding Institute, and Vegetable Breeding Research Center, College of Agriculture and Life Sciences, Seoul National University, Seoul 08826, Korea; bk54@snu.ac.kr

**Keywords:** γ-ray, mutation, frequency, spectrum, chimera, stability, *Cymbidium*

## Abstract

Ionizing radiation combined with in vitro tissue culture has been used for development of new cultivars in diverse crops. The effects of ionizing radiation on mutation induction have been analyzed on several orchid species, including *Cymbidium*. Limited information is available on the comparison of mutation frequency and spectrum based on phenotypes in *Cymbidium* species. In addition, the stability of induced chimera mutants in *Cymbidium* is unknown. In this study, we analyzed the radiation sensitivity, mutation frequency, and spectrum of mutants induced by diverse γ-ray treatments, and analyzed the stability of induced chimera mutants in the *Cymbidium* hybrid cultivars RB003 and RB012. The optimal γ-irradiation conditions of each cultivar differed as follows: RB003, mutation frequency of 4.06% (under 35 Gy/4 h); RB012, 1.51% (20 Gy/1 h). Re-irradiation of γ-rays broadened the mutation spectrum observed in RB012. The stability of leaf-color chimera mutants was higher than that of leaf-shape chimeras, and stability was dependent on the chimera type and location of a mutation in the cell layers of the shoot apical meristem. These results indicated that short-term γ-irradiation was more effective to induce mutations in *Cymbidium*. Information on the stability of chimera mutants will be useful for mutation breeding of diverse ornamental plants.

## 1. Introduction

The Orchidaceae is among the largest families of angiosperms, and is composed of approximately 736 genera and about 28,000 species [1]. Among the diverse genera in the family, *Dendrobium*, *Phalaenopsis*, *Oncidium*, and *Cymbidium* are important floricultural crops in Asian countries [2]. In particular, *Cymbidium* is economically important in northeastern Asia, including Korea, China, and Japan [3]. *Cymbidium* species are conveniently divided into two groups on the basis of the native habitat and climate region, i.e., temperate and subtropical or tropical regions [4,5]. There is continuous demand for development of new *Cymbidium* cultivars on account of the attractiveness of the foliage as well as the flower colors and fragrance. To date, many *Cymbidium* cultivars have been developed via natural selection or artificial cross-breeding, although transformation has also been applied in *Cymbidium* breeding [6]. However, development of a new *Cymbidium* cultivar by means of cross-breeding is time-consuming because of the long vegetative growth stage.

Mutation breeding techniques using physical mutagens (e.g., γ-rays, X-rays, and ion particles) or chemical mutagens (e.g., ethyl methanesulfonate, *N*-nitroso-*N*-methylurea, and colchicine) have been widely used to develop mutant cultivars in diverse plant species, including food and ornamental crops. In 226 plant species, 3308 mutants, including two *Cymbidium* mutants, that were predominantly (77.5%) developed using physical mutagens have been registered in the Mutant Variety Database of the joint Food and Agriculture Organization of the United Nations/International Atomic Energy Agency [7]. Among the diverse ornamental plants registered on the database, the most numerous mutants are those of chrysanthemum (*Chrysanthemum* spp., 274 mutants) followed by rose (*Rosa* spp., 67 mutants), dahlia (*Dahlia* spp., 36 mutants), alstroemeria (*Alstroemeria* spp., 35 mutants), streptocarpus (*Streptocarpus* spp., 30 mutants), and carnation (*Dianthus caryophyllus*, 28 mutants) [7]. The combination of treatment with a physical mutagen and in vitro tissue culture has been used to shorten the breeding period of orchids [8,9]. Thus, integration of γ-irradiation and in vitro tissue culture may be an efficient procedure for development of mutant cultivars in *Cymbidium*.

The treatment of seeds, buds (tip/node cuttings), callus, and rhizomes with a mutagen induces chimeras in M_1_ plants because mutations are induced in individual cells and regenerated shoots are recovered from pre-existing multicellular meristems [10]. In seed-propagated plants, the induced chimera is commonly dissociated by selfing of M_1_ plants, whereas, in vegetatively propagated plants chimera dissociation by selfing is of limited use because selfing results in loss of the desirable original characteristics. Geier [10] suggests that plant regeneration via adventitious buds or somatic embryos, which permits the genetic background to be retained, may be useful to dissociate the chimera in vegetatively propagated plants. In the majority of angiosperms, the shoot apical meristem (SAM) is composed of three layers: the outer meristem layer (L1), the second meristem layer (L2), and the inner corpus (L3) [11,12]. Mutagen-treated plants are composed of heterogeneous cells in the three layers, which can be categorized into three chimera types: sectorial chimeras, which have an unstable heterogenomic population of cells traversing more than one layer of the SAM; mericlinal chimeras, which have an unstable heterogenomic population of cells within a single layer of the SAM; and periclinal chimeras, which have a stable, uniform, and genetically distinguished layer of cells of the SAM [10,12]. The phenotypes of mutants are diverse according to the type and extent of these chimeras. Thus, an understanding of chimerism is necessary to develop a new *Cymbidium* mutant cultivar by mutagenesis.

In the present study, we evaluated the optimal γ-ray dose to induce mutations in *Cymbidium*, and compared the mutation frequency and spectrum of leaf mutants induced by diverse γ-ray treatments. In addition, we investigated the stability of chimera mutants using plants secondarily regenerated from selected rhizomes and the initially regenerated mutant plant.

## 2. Results

### 2.1. Effects of γ-Irradiation on Rhizome Growth Parameters

**Figure 1:**
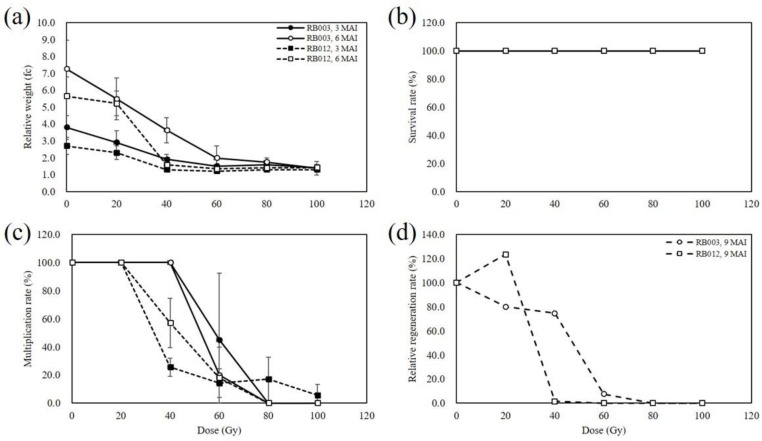
Relative weight, survival, multiplication, and relative regeneration rate of *Cymbidium* hybrids, RB003 and RB012 rhizomes at 3, 6, and 9 months after γ-irradiation. (**a**) Relative weight of rhizomes; (**b**) survival rate of rhizomes; (**c**) multiplication rate of rhizomes; (**d**) relative regeneration rate of rhizomes. Relative weight, survival, and multiplication rate of rhizomes were measured at 3 and 6 months after irradiation, whereas the relative regeneration rate was analyzed at 9 months. fc, fold-change. MAI, months after irradiation. Error bars indicate the standard error of the mean (n = 5).

At 3 months after γ-irradiation, the relative weight of the γ-irradiated rhizomes of the cultivars RB003 and RB012 gradually decreased with increase in γ-ray dose. At 6 months after γ-irradiation, the responses of rhizomes to γ-irradiation were more distinct; rhizome growth was strongly inhibited at γ-ray doses of more than 60 and 40 Gy in RB003 and RB012, respectively (Figure 1a). All γ-irradiated rhizomes of the two cultivars survived at 3 and 6 months after irradiation (Figure 1b). However, at 3 months after γ-irradiation the multiplication rate of the γ-irradiated rhizomes rapidly decreased at doses of more than 60 and 40 Gy in RB003 and RB012, respectively. In addition, at 6 months after γ-irradiation, multiplication was not observed at doses of more than 80 Gy in the two cultivars (Figure 1c). The γ-irradiated rhizomes were regenerated from about 8 months after irradiation in the two cultivars, although regeneration of the control was detected from 7 months after initial culture. Therefore, the relative regeneration rate of γ-irradiated populations was evaluated at 9 months after irradiation. The regeneration of RB003 rhizomes decreased in a dose-dependent manner and strongly inhibited regeneration was observed at doses of more than 60 Gy. However, RB012 rhizomes irradiated with 20 Gy showed a higher rate of regeneration compared with that of the control, and at doses exceeding 40 Gy regeneration was strongly suppressed (Figure 1d).

Given that all γ-irradiated populations survived during the first 6 months after treatment, it was impossible to estimate the 50% lethal dose (LD_50_) for the two cultivars (Figure 1b). At 3 months after γ-irradiation of rhizomes, the 50% reduction dose (RD_50_) for the two cultivars was estimated as follows: RB003, 30.0 Gy (based on the relative weight) and 58.2 Gy (based on the multiplication rate); and RB012, 29.0 Gy (based on the relative weight) and 33.5 Gy (based on the multiplication rate). At 6 months, the estimated RD_50_ for each cultivar was as follows: RB003, 34.4 Gy (based on the relative weight) and 52.5 Gy (based on the multiplication rate); and RB012, 30.6 Gy (based on the relative weight) and 44.1 Gy (based on the multiplication rate) (Figure 1a,c). At 9 months, the RD_50_ based on the relative regeneration rates for each cultivar was 47.4 Gy and 34.4 Gy for RB003 and RB012, respectively (Figure 1d).

### 2.2. Comparison of Mutation Frequency and Spectrum among γ-Irradiated Populations

In the RB003 populations, the mutation frequency was elevated with an increase in γ-ray dose rate as follows: 50 Gy/24 h, mutation frequency of 0.35%; 50 Gy/16 h, 0.81%; and 50 Gy/8 h, 3.73%. In addition, the two populations γ-irradiated with 50 Gy/8 h and 35 Gy/4 h showed the same mean regenerants per bottle of 3.11 as well as similar mutation frequencies: 50 Gy/8 h, mutation frequency of 3.73%; and 35 Gy/4 h, 4.06%. Under these irradiation conditions, the mutation frequency and spectrum were the highest observed among the γ-irradiated RB003 populations (Table 1).

In the RB012 populations, the mean regenerants per bottle decreased precipitously with increase in γ-ray dose rate as follows: 40 Gy/24 h, mean regenerants per bottle of 4.63; 40 Gy/16 h, 1.82; and 40 Gy/8 h, 0.11. The two populations γ-irradiated with 40 Gy/24 h and 20 Gy/1 h showed similar mean regenerants per bottle (4.63 and 4.36, respectively) as well as similar mutation frequencies: 40 Gy/24 h, mutation frequency of 1.42%; and 20 Gy/1 h, 1.51%. The highest mutation frequency observed was 3.24% in the irradiation condition 30 Gy/4 h; however, it is impossible to consider this is to be the optimal irradiation condition because of the severely reduced regeneration. The highest mutation spectrum was observed in the population γ-irradiated with 20 Gy/1 h, which also showed the second-highest mutation frequency of 1.51% (Table 1). In the re-irradiated RB012 population, regeneration was reduced by 42.4% after re-irradiation (30–30 Gy/24 h) of the RB012 population initially γ-irradiated with 30 Gy/24 h (Table 1). Compared with the RB012 population γ-irradiated with 30 Gy/24 h, the mutation spectrum of the re-irradiation (30–30 Gy/24 h) population was broader, although no difference in mutation frequency was observed (Table 1).

**Figure 2:**
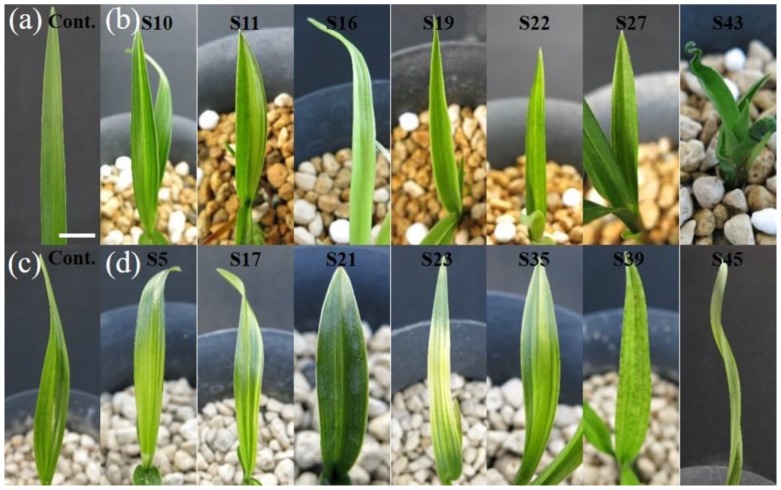
Diverse leaf-color or -shape mutants derived from *Cymbidium* hybrids RB003 and RB012. (**a**) RB003 control; (**b**) leaf mutants derived from RB003; (**c**) RB012 control; (**d**) leaf mutants derived from RB012. (**b**) indicates S10, S11, S16, S19, S22, S27 (leaf-color mutants), and S43 (leaf-shape mutant), while (**d**) indicates S5, S17, S21, S23, S35, S39 (leaf-color mutants), and S45 (leaf-shape mutant). Scale bar: 1 cm.

In the γ-irradiated populations of the two cultivars, diverse leaf-color or -shape mutants were observed and the proportion of leaf-color mutants was higher than that of leaf-shape mutants (Table 1, Figure 2).

### 2.3. Evaluation of Stability among Leaf Mutants

**Figure 3:**
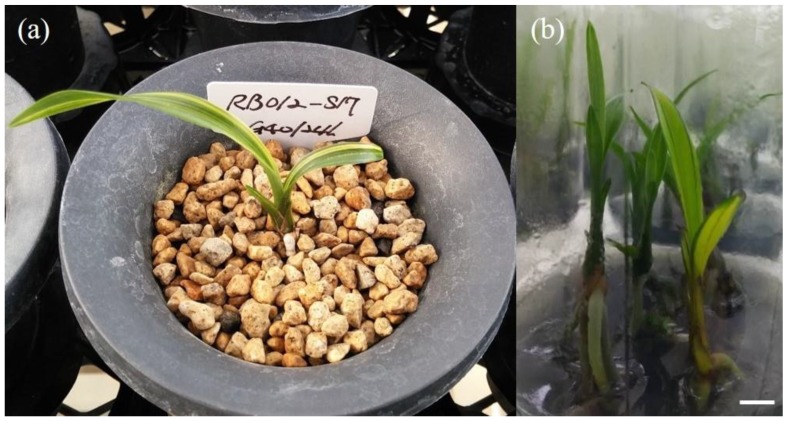
Segregation in the selected mutant RB012-S17. (**a**) The first mutant plant regenerated from γ-irradiated rhizomes; (**b**) segregation of newly regenerated plants from the first selected rhizomes in the M_1_V_1_ generation. Scale bar: 1 cm.

A total of 101 leaf-color or -shape mutants (52 mutants derived from RB003 and 49 from RB012) were tested to analyze the stability of the induced chimera mutants (Table 2). The chimera stability of the first selected mutant plant induced by regeneration of γ-irradiated rhizomes was higher than that of the rhizome closely attached to the mutant plant: RB003, plant (43.4%), rhizome (33.3%); and RB012, plant (33.3%), rhizome (18.6%). The leaf-color mutants were observed to be relatively stable chimera types, but the leaf-shape mutants were unstable in the two cultivars. The most stable chimera type was the yellow marginal stripe types, which showed stability of 50.0% (from the rhizome) and 83.3% (from the plant) (assumed to be a periclinal chimera, a mutation of the L2 layer of the SAM), followed by: yellow broad stripe types with stability of 50.0% (rhizome) and 66.6% (plant) (assumed to be a periclinal chimera, a mutation of the L3 layer of the SAM); yellow narrow stripe types with stability of 25.5% (rhizome) and 100% (plant) (assumed to be a mericlinal chimera, a mutation of the L3 layer); bright green types with stability of 25.0% (rhizome) and 50.0% (plant) (assumed to be a sectorial chimera, a mutation of the L2 and L3 layers); yellow large spot types with stability of 12.5% (rhizome) and 12.5% (plant); and comb types with stability of 0% (rhizome) and 33.3% (plant) (assumed to be a mericlinal chimera, a mutation of the L3 layer) (Table 2). In the case of the selected mutant RB012-S17, the phenotype of the first selected mutant plant induced by regeneration of γ-irradiated rhizomes with 40 Gy/24 h was of the yellow narrow stripe type; however, newly regenerated in vitro plants from the rhizome closely attached to the mutant plant segregated in the M_1_V_1_ generation, and the yellow broad stripe type ultimately selected was stabilized in the M_1_V_5_ generation (Figure 3).

## 3. Discussion

### 3.1. Optimal γ-Irradiation Condition for Mutation Induction

In plant mutation breeding, the optimal irradiation condition is considered to be the most important factor to induce desirable mutants with the minimum collateral DNA damage. Irradiation dose has been mainly used for determination of the optimal irradiation condition in diverse plant species, including orchids [5,8,13,14,15,16,17,18]. However, the optimal doses suggested by previous researchers are diverse: e.g., LD_10_ in rice seeds [18]; LD_30–50_ and RD_30–50_ in crop seeds [19]; LD_20–30_ in in vitro tissues [20]; and RD_50_ in *Cymbidium* protocorm-like bodies (PLBs) [9]. Furthermore, irradiation duration and dose rate, a complex concept of dose and duration, are also important factors for induction of mutations. The effect of dose rate on mutation induction has varied substantially in previous researches. A low dose rate was reported to be more effective to induce mutations than a high dose rate in barley and maize [21]. However, Mabuchi and Matsumura [22] suggested that a high dose rate could induce a higher mutation frequency than a low dose rate under an identical total dose in maize. In contrast, the mutation frequency is dependent on the total dose, not the dose rate, in chrysanthemum [23]. Recently, Kim et al. [24] proposed that a specific irradiation duration could induce a higher mutation frequency and broader spectrum of mutants in chrysanthemum. In the present study, we used the RD_50_ based on the multiplication and relative regeneration rates as a guide to construct mutant populations from two *Cymbidium* cultivars (Figure 1).

### 3.2. Frequency and Spectrum of Induced Leaf Mutants

The frequency of somatic mutations is extremely low and differs considerably among plant species. In Arabidopsis, the frequency of somatic mutations was calculated as approximately 1.6 × 10^−10^ mutations per base per cell division [25]. Somaclonal variants were observed at frequencies of 0.6% and 0.05% from tissue culture of *Pelargonium* spp. and a *Cymbidium* hybrid, respectively [4,26]. Luan et al. [8] analyzed the LD_50_, mutation frequency, and spectrum of mutants induced by γ-ray and 320 MeV ^12^C^6+^ ion irradiations in two *Paphiopedilum* species. Several leaf-color (white margin and chlorophyll variegation) or -shape (large leaves, narrow leaves, and enormous shoot buds) mutants were observed with a mutation frequency of 3% in the two populations irradiated with 320 MeV ^12^C^6+^ ions of 3 Gy, whereas no mutants were observed in the γ-irradiated populations. In addition, the proportions of leaf-color to leaf-shape mutants were 12:1 and 1:11 in the *P. delenatii* and *P. callosum* populations, respectively [8]. In two *Dendrobium* species, a chlorophyll variegated mutant (induced by irradiation with 0.4 Gy) and leaf-shape mutants (irradiation with 0.2–2.0 Gy) were identified in the ^12^C^6+^ ion-irradiated *D. mirbellianum* and *D. crumenatum* populations, respectively [13]. Ahmad et al. [14] reported only leaf-shape mutants (narrow and pointed leaves, and abnormally shaped leaves) in an *Oncidium lanceanum* population irradiated with 220 MeV ^12^C^5+^ ion of 1–2 Gy. However, compared with previous studies, we identified a relatively higher proportion of leaf-color mutants and a broader spectrum of mutants in the present study (Table 1, Figure 2). This finding may be due to the differences in plant species or radiation type used. Furthermore, Prina et al. [27] reported that treatment of homozygous barley seeds with mutagens resulted in two types of chlorophyll variegations (light green, ~70%; and albino), whereas treatment of heterozygous seeds with mutagens dramatically changed the spectrum of somatic-sector mutations. In this respect, the high heterozygosity of the two *Cymbidium* cultivars, which are hybrids of *C. sinense* and *C. goeringii*, used in the current study also may be a cause of the broad mutation spectrum.

### 3.3. Effects of Short-Term Irradiation and Re-irradiation on Mutation Induction

Kim et al. [24] reported that at an identical total γ-ray dose of 30 Gy, an irradiation duration of 4 h among durations of 1, 4, 8, 16, and 24 h induced the highest frequency of flower-color mutations in chrysanthemum. Kodym et al. [19] suggested that radiation-induced DNA damage is restored by a repair mechanism mainly in the S phase of the DNA replication cycle and that a long duration of irradiation reduces the effects of the radiation dose. In the present study, the mutation frequency increased with decrease in irradiation duration in the 50 Gy-irradiated RB003 populations and the mutation frequency of the RB012 population irradiated with 20 Gy/1 h was similar to that of the population γ-irradiated with 40 Gy/24 h. In addition, the highest mutation frequency was observed with irradiation durations of 4 h and 1 h in the RB003 and RB012 populations, respectively (Table 1). These results indicate that short-term irradiation is more effective to induce mutations than long-term irradiation in *Cymbidium*. Kodym et al. [19] reported that recurrent irradiation treatment was conducted to broaden the mutation spectrum and to increase the chances of obtaining desirable mutants in diverse plant species, but the experiments did not yield the expected results. However, several studies have reported the effectiveness of re-irradiation of ion particles in ornamental flower species [28]: e.g., cyclamen [29], *Osteospermum* spp. [30], and chrysanthemum [31]. In the present study, the mutation spectrum was broadened by re-irradiation with γ-rays, although no increase in the mutation frequency was observed and the size of the re-irradiated population was larger than that of single-irradiated population (Table 1).

### 3.4. Stability of Induced Chimera Mutants

Mutagens such as γ-rays have been applied to increase the mutation frequency and lead to genetically heterogeneous cells, which is a chimeric condition. Chimera types are determined by the mode of spread, spatial arrangement, and competitiveness of a mutated cell among the mutated and wild-type cells in the SAM layers [10]. In vegetatively propagated plants, Geier [10] suggested several practical methods for chimera dissociation, such as mechanical wounding, application of plant hormones, lateral bud sprouting, in vitro shoot proliferation, adventitious shoot regeneration, and somatic embryogenesis. In the present study, periclinal chimera types (yellow marginal stripe (assumed to be a mutation in the L2 layer) and yellow broad stripe (L3)) were more stable than mericlinal chimera types (yellow narrow stripe (L3) and comb (L3)) (Table 2), which is consistent with previous reports [10]. In a previous study, the stability of selected PLBs, which produced a somaclonal variant with a yellow marginal stripe, was 40% in a *Cymbidium* hybrid [4]. Yamaguchi et al. [17] reported that the flower-color mutants induced by γ-rays were all identified as periclinal chimeras, but those induced by carbon-ion irradiations displayed a higher proportion of solid type mutants in chrysanthemum. Although with regard to chimera dissociation carbon ions are advantageous to γ-rays, with respect to horticultural potential γ-rays may be more useful than carbon ions due to the inducible phenotypic diversity. Prina et al. [27] noted that positional variegation is caused by differential gene expression, which is not a chimeric condition. In the present study, the phenotype of mutants showing yellow large spots was unstable in the next generation, which may be a result of differential gene expression (Table 2). We observed relatively higher stability of chimeras on the plant than the rhizome. However, we suggest that selection of stable mutant rhizomes through recurrent selection is more appropriate than selection of regenerated mutant plants, which may increase the risk of contamination or death during meristem culture, a long propagation period, and unintended rearrangement of cell layers. Given that the present results on mutation frequency and spectrum, and stability, were derived from diverse populations γ-irradiated only once and from the M_1_V_1_ generation, respectively, repetition of the experiment and comparison of stability in later generations are required to be able to draw more precise conclusions. Nevertheless, the present results provide useful information for mutation breeding of vegetatively propagated crops such as *Cymbidium* and for an improved understanding of chimerism in monocotyledons.

## 4. Materials and Methods

### 4.1. Plant Materials and Tissue Culture Procedure

Two *Cymbidium* hybrid (*C. sinense* × *C. goeringii*) cultivars, RB003 and RB012, were used in this study (Figure S1). Tissue culture procedure followed the method of Shin et al. [32] with slight modifications. Rhizomes of the two cultivars were cultured at 24 ± 1 °C under a 16-h photoperiod provided by white fluorescent light (photosynthetic photon flux density = 50 μmol m^−2^ s^−1^) on medium (pH 5.35) comprising 0.2% Hyponex (N:P:K = 6.5:6:19; Hyponex Japan Co., Ltd., Osaka, Japan), 0.1% Hyponex (N:P:K = 20:20:20), 3% sucrose (Duchefa B.V., Haarlem, The Netherlands), 0.3% peptone (Duchefa B.V.), 0.075% activated charcoal (Sigma-Aldrich, St Louis, MO, USA), and 0.38% plant agar (Duchefa B.V.).

### 4.2. Optimal γ-Ray Dose Determination

Rhizomes of the two cultivars were irradiated with six doses of γ-rays (0, 20, 40, 60, 80, or 100 Gy) emitted from a ^60^Co source (150 TBq capacity; AECL, Chalk River, ON, Canada) for 24 h at the Korea Atomic Energy Research Institute, Jeongeup, Korea. The γ-irradiated rhizomes were immediately transferred to fresh culture medium. The growth of γ-irradiated rhizomes was evaluated based on four growth parameters. Relative weight, survival, and multiplication rate of rhizomes were measured at 3 and 6 months after irradiation (Figure S2), whereas the relative regeneration rate was analyzed at 9 months. The γ-irradiated rhizomes were subcultured at 3-month intervals. The experiments were performed with five biological replicates, with seven rhizomes per replicate.

### 4.3. Mutant Population Construction

A previous study revealed that the RD_50_ of *Cymbidium* PLBs for γ-irradiation duration of 24 h was reduced with decrease in irradiation duration as follows: 40.0 Gy (for irradiation duration of 24 h), 37.9 Gy (16 and 8 h), 23.6 Gy (4 h), and 16.1 Gy (1 h) [33]. Therefore, based on the RD_50_ values calculated from the multiplication and relative regeneration rates for an irradiation duration of 24 h, γ-irradiated populations of the two cultivars were constructed using suggested doses for each irradiation duration of 1, 4, 8, and 16 h. Mutant populations of the two cultivars were constructed using diverse γ-irradiation conditions as follows: RB003, irradiation conditions of 50 Gy/24 h, 50 Gy/16 h, 50 Gy/8 h, 35 Gy/4 h, and 25 Gy/1 h; RB012, irradiation conditions of 40 Gy/24 h, 40 Gy/16 h, 40 Gy/8 h, 30 Gy/4 h, 20 Gy/1 h, 30 Gy/24 h, and 30–30 Gy/24 h (re-irradiation after mutant selection on the population treated with 30 Gy/24 h). The experiments for each γ-irradiation condition were performed with about 40 and 50 culture bottles (seven rhizomes per culture bottle) for RB003 and RB012, respectively, except the irradiation condition of 30–30 Gy/24 h on RB012, which was conducted with about 200 culture bottles.

### 4.4. Phenotype and Stability Analysis of Leaf Mutants

Phenotype analysis of the mutant populations derived from RB003 and RB012 was conducted at three time points until 10 and 12 months after γ-ray treatment, respectively. Mutation frequency and spectrum were calculated as the percentage of mutants relative to the total regenerated plants and the type of mutants, respectively. Data for regeneration and mutation frequency were analyzed using Duncan’s multiple range test at a significance level of 0.05 using IBM-SPSS Statistics 17.0 software (IBM, Armonk, NY, USA). The stability of mutants was analyzed on newly regenerated plants from two selected mutant tissues: the mutant plant regenerated from γ-irradiated rhizomes and its closely attached rhizome. If the newly regenerated M_1_V_1_ plant showed characteristics identical to those of the M_1_V_0_ mutant plant, the M_1_V_0_ tissue was classified as a putative stable mutant.

## Figures and Tables

**Table 1:** Regeneration, mutation frequency, and spectrum of leaf mutants derived from diverse γ-ray treatments in the *Cymbidium* hybrids RB003 and RB012.

Total Dose/Irradiation Duration	No. of Regenerants per Cultured Bottle	No. of Mutants (%)	Description of Mutants (No. of Mutants)		Mutation Spectrum
			Leaf Color	Leaf Shape	
RB003					
Control	5.42a^a^	0 (0.00a^b^)	-	-	-
50 Gy/24 h	2.50b	1 (0.35a)	Comb/yellow broad stripe (1)	-	1
50 Gy/16 h	3.04ab	3 (0.81a)^c^	Comb (1), bright green/yellow large spot (1), yellow marginal stripe (1)	Wrinkle (1)	4
50 Gy/8 h	3.11ab	11 (3.73b)	Bright green/yellow large spot (1), bright green (2), comb (3), comb/yellow broad stripe (1), bright green/comb (1),	Abnormal (2), dwarf (1), wrinkle (4)	8
35 Gy/4 h	3.11ab	14 (4.06b)	Yellow marginal stripe (1), bright green (2), comb/yellow broad stripe (1), yellow marginal/narrow stripe (1), bright green/yellow large spot (3)	Wrinkle (5), dwarf (1)	7
25 Gy/1 h	3.42ab	7 (2.02ab)	Bright green/comb (2), bright green/yellow large spot (2), yellow marginal stripe (1)	Abnormal (2)	4
RB012					
Control	16.67a	0 (0.00a)	-	-	-
40 Gy/24 h	4.63bc	9 (1.42b)	Yellow narrow stripe (1), yellow large spot (4), snakeskin (2), yellow marginal stripe (2),	-	4
40 Gy/16 h	1.82cd	1 (0.23a)	Yellow marginal stripe (1)	-	1
40 Gy/8 h	0.11d	0 (0.00a)	-	-	-
30 Gy/4 h	0.65d	3 (3.24c)	Yellow marginal stripe (2), yellow large spot (1)	-	2
20 Gy/1 h	4.36bc	10 (1.51b)	Yellow marginal stripe (2), yellow large spot (3), bright green (1), snakeskin (2), yellow narrow stripe (1), yellow broad stripe (1)	-	6
30 Gy/24 h	7.14b	4 (0.68a)	Yellow large spot (1), yellow marginal stripe (2)	Abnormal (1)	3
30–30 Gy/24 h	4.11c	21 (0.67a)	Yellow narrow stripe (1), yellow marginal stripe (6), yellow broad stripe (3), bright green (1), yellow large spot (5), snakeskin (1),	Wrinkle (1), dwarf (1), abnormal (2)	9

^a, b^ Values are means (n = 3). Mean values followed by different lower-case letters within each column for each treatment were significantly different at *p* ≤ 0.05 using Duncan’s multiple range test; ^c^ A mutant showing more than two characteristics was counted as only one.

**Table 2:** Stability of leaf mutants induced by γ-irradiation in the *Cymbidium* hybrids, RB003 and RB012 populations.

Characteristic	No. of Mutants	Stability (%)	
		Rhizome^a^	Plant^b^
RB003			
Yellow marginal stripe, color	3	-	3/3 (100.0)
Yellow broad stripe, color	3	-	1/2 (50.0)
Yellow narrow stripe, color	1	-	1/1 (100.0)
Bright green, color	15	2(2^c^)/6 (33.3)	3/6 (50.0)
Yellow large spot, color	7	2/2 (100.0)	1/2 (50.0)
Comb, color	7	0/1 (0.0)	1/3 (33.3)
Dwarf, shape	2	-	-
Wrinkle, shape	10	0/3 (0.0)	0/5 (0.0)
Abnormal, shape	4	-	0/1 (0.0)
Total	52	4(2)/12 (33.3)	10/23 (43.4)
RB012			
Yellow marginal stripe, color	14	5(2)/10 (50.0)	2/3 (66.7)
Yellow broad stripe, color	4	2(1)/4 (50.0)	1/1 (100.0)
Yellow narrow stripe, color	4	1(2)/4 (25.0)	2/2 (100.0)
Bright green, color	2	0/2 (0.0)	-
Yellow large spot, color	15	0/14 (0.0)	0/6 (0.0)
Snakeskin, color	5	0/5 (0.0)	-
Dwarf, shape	1	0/1 (0.0)	-
Wrinkle, shape	1	0/1 (0.0)	0/1 (0.0)
Abnormal, shape	3	0/2 (0.0)	0/2 (0.0)
Total	49	8(5)/43 (18.6)	5/15 (33.3)

^a^ Rhizome closely attached to the selected mutant plant; ^b^ Mutant plant regenerated from γ-irradiated rhizomes; ^c^ Segregation.

## Supplementary Materials

The following are available online at https://www.mdpi.com/2223-7747/9/4/546/s1. Figure S1: The phenotypes of the two *Cymbidium* hybrids, RB003 and RB012, used for this study. Figure S2: Growth response of rhizomes of the *Cymbidium* hybrids, RB003 and RB012 at 3 and 6 months after γ-irradiation.
