# Supplementary material for: Frequency, Spectrum, and Stability of Leaf Mutants Induced by Diverse γ-Ray Treatments in Two Cymbidium Hybrids
Source: Plants (Basel). 2020 Apr 23;9(4):546. doi: 10.3390/plants9040546 (PMC7238856; doi:10.3390/plants9040546)
Supplement: Supplementary file 1 [file plants-09-00546-s001.pdf]

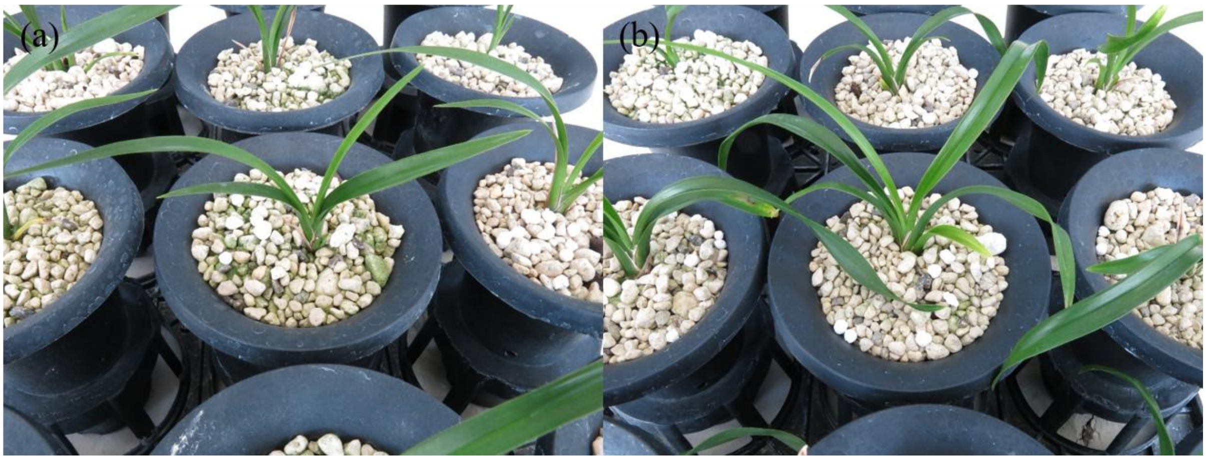

Figure S1. The phenotypes of the two *Cymbidium* hybrids, RB003 and RB012, used for this study. (**a**) RB003; (**b**) RB012.

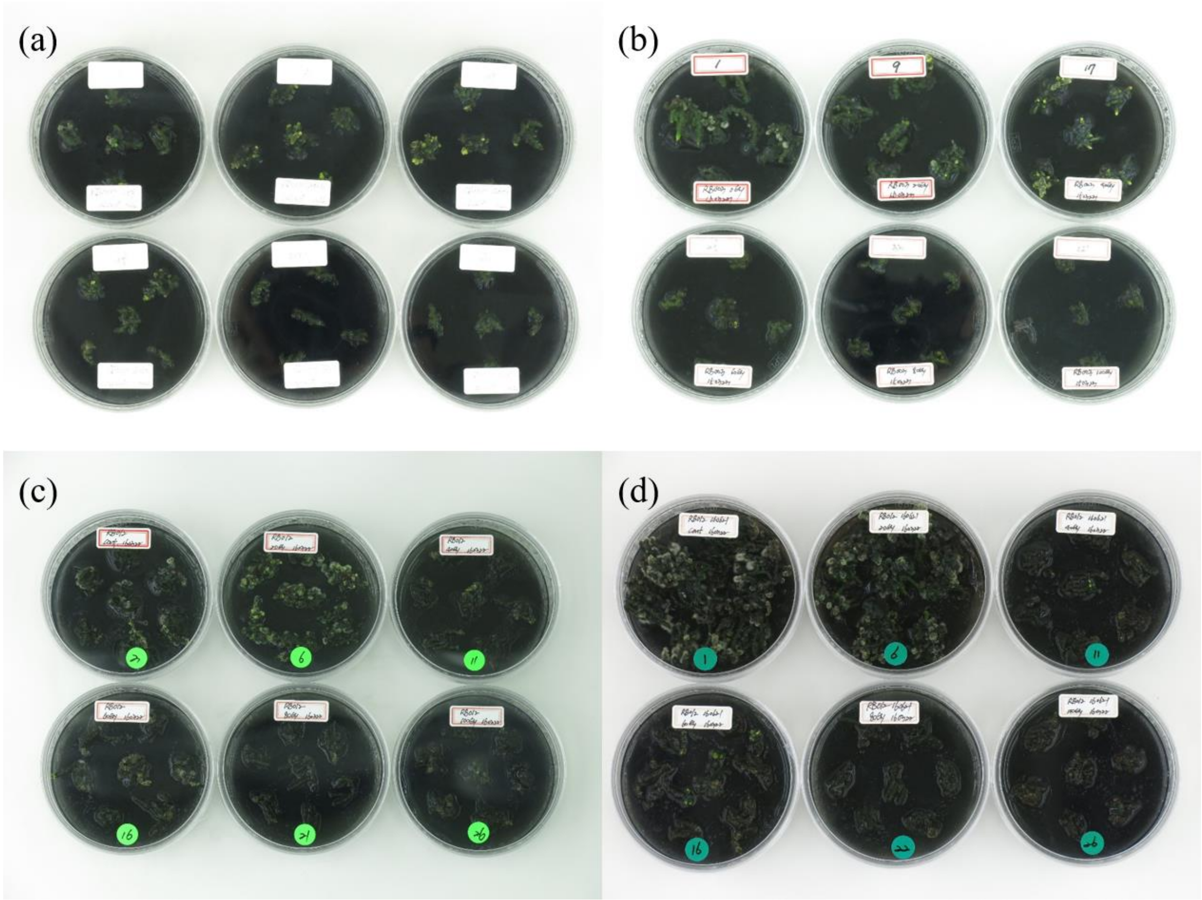

Figure S2. Growth response of rhizomes of the *Cymbidium* hybrids, RB003 and RB012 at 3 and 6 months after γ-irradiation. (**a**) RB003, 3 months after γ-irradiation; (**b**) RB003, 6 months after γ-irradiation; (**c**) RB012, 3 months after γ-irradiation; (**b**) RB012, 6 months after γ-irradiation. From the upper left: 0, 20, 40, 60, 80, and 100 Gy treatments.
